# A Report by the Editor-in-Chief for *Molecular Biology and Evolution* (MBE), Volume 37

**DOI:** 10.1093/molbev/msab319

**Published:** 2021-11-13

**Authors:** Sudhir Kumar

**Affiliations:** Institute for Genomics and Evolutionary Medicine, Temple University, Philadelphia, PA, USA

*Molecular Biology and Evolution* is a proudly society-owned journal, providing members of the Society for Molecular Biology and Evolution (SMBE) with a high-impact outlet for their research and opportunities for active participation in scientific publishing.

The world faced many challenges in 2020, which were shared by our community. We are particularly grateful to our editors and reviewers, who continued their efforts to move science forward in the face of disrupted work environments and urgent personal commitments.

The *MBE* community also prepared for a major change in 2020, transitioning from a hybrid publishing model to fully open-access publishing. This transition required modifications to the submission and production of manuscripts and the publication fee structure. By August 2020, all submissions were required to be evaluated under an open-access license, allowing the January 2021 issue to contain only open-access manuscripts. With this development, *MBE* content became accessible to readers everywhere without paywalls. Although the changes in publication fees raised concerns about the accessibility of publication to researchers lacking sufficient financial support, *MBE* maintains its mission to support the publication of novel and high-impact research regardless of the authors’ ability to pay.

The transition to full open access also marked the end of print publication for *MBE*. The December 2020 issue was *MBE*’s last regular volume printed on paper. *MBE* continues to highlight author-contributed original artwork on our website, with a virtual cover image selected for each issue.

During the 2020 calendar year, *MBE* received 1,247 original submissions and 482 revised submissions from authors based in 54 countries. Although more than 90% of the submissions came from Europe, Asia, and North America, the proportional contribution among these regions continues to shift, with the proportion of manuscripts submitted by authors based in Asia (33%) narrowly surpassing the proportion submitted by authors based in Europe. Authors based in North America submitted 26% of original manuscripts received by *MBE* in 2020. Articles account for almost 92% of original submissions, followed by Letters (7%). *MBE* continues to publish Brief Communications (usually to communicate software and resource updates), Perspectives, and Reviews in smaller numbers, as well as Protocols by invitation. Press articles, written by *MBE*’s Press Officer Joseph Caspermeyer, highlight articles expected to generate broad interest among nonspecialist readers.

*MBE*’s impact factor for 2020 (citations during 2020 of manuscripts published in 2018 and 2019) is 16.240. Citation trends thus far in 2021 suggest that next year’s impact will remain strong.

*MBE*’s rejection rate for original submissions in 2020 was 73%, with nearly 57% of these rejected without soliciting external review. Of manuscripts rejected at this stage, 30% were offered an immediate transfer to our sister journal Genome Biology and Evolution (*GBE*). Rejection prior to external peer-review is meant to save time for authors and reviewers in finding the most suitable venue for a given manuscript. This procedure helped us keep the average time until the first decision to 23 days for manuscripts submitted in 2020. The average time to reach a final decision for a research article was 36 days, an improvement in speed from 2019, despite our impressions that everything was moving slower in “pandemic time.”

In ongoing efforts to work more closely with *GBE*, *MBE* has experimented with changes to our editorial process that expedite the transfer of high-quality manuscripts suitable for publication in *GBE*. As part of the routine manuscript evaluation, handling editors assess whether manuscripts would be potentially suitable for publication in *GBE*, regardless of their outcome at *MBE*. Any manuscripts deemed potentially suitable for *GBE* but not chosen as a high priority for publication in *MBE* are offered transfer to *GBE* in their decision letter. Of manuscripts submitted in 2020, 373 were given an option to transfer to *GBE*, including 216 immediate transfers and 157 after external peer-reviews at *MBE*. More than half of these manuscripts had positive outcomes at *GBE*, i.e., they were either accepted or a revision was invited. The offers of transfer to *GBE* had a response rate less than 50%, but ∼58% of respondents did elect to transfer their manuscript to *GBE*. Although these results are generally encouraging, *MBE* will continue to work with the editors and staff at *GBE* to make the transfer process more appealing to authors and improve *MBE* editors’ ability to identify articles that will be successful at *GBE*.

Although the global pandemic removed our ability to convene our editorial board in person in Quebec City, our virtual editorial board meetings were well-attended. We are grateful to our editors’ service to the SMBE community and thankful to the editorial staff for supporting all the editors and authors.

